# A novel nonsense mutation in *ARMC5* causes primary bilateral macronodular adrenocortical hyperplasia

**DOI:** 10.1186/s12920-021-00896-0

**Published:** 2021-05-10

**Authors:** Wen-Tao He, Xiong Wang, Wen Song, Xiao-Dong Song, Yan-Jun Lu, Yan-Kai Lv, Ting He, Xue-Feng Yu, Shu-Hong Hu

**Affiliations:** 1 Branch of National Clinical Research Center for Metabolic Disease, Hubei, Department of Endocrinology, Tongji Hospital, Tongji Medical College, Huazhong University of Science and Technology, Wuhan, 430030 China; 2 Department of Laboratory Medicine, Tongji Hospital, Tongji Medical College, Huazhong University of Science and Technology, Wuhan, 430030 China; 3 Department of Urology, Tongji Hospital, Tongji Medical College, Huazhong University of Science and Technology, Wuhan, 430030 China; 4 Department of Pathology, Tongji Hospital, Tongji Medical College, Huazhong University of Science and Technology, Wuhan, 430030 China

**Keywords:** PBMAH, ARMC5, Cushing’s syndrome, Genetic diagnosis, Case report

## Abstract

**Background:**

Primary bilateral macronodular adrenocortical hyperplasia (PBMAH) is a rare form of adrenal Cushing’s syndrome. The slowly progressing expansion of bilateral adrenal tissues usually persists for dozens of years, leading to delayed onset with severe conditions due to chronic mild hypercortisolism. About 20–50% cases were found to be caused by inactivating mutation of armadillo repeat-containing protein 5 (*ARMC5*) gene.

**Case presentation:**

A 51-year-old man was admitted for severe diabetes mellitus, resistant hypertension, centripedal obesity and edema. PBMAH was diagnosed after determination of adrenocorticotropic hormone and cortisol levels, dexamethasone suppression tests and abdominal contrast-enhanced CT scanning. The metabolic disorders of the patient remarkably improved after sequentially bilateral laparoscopic adrenalectomy combined with hormone replacement. Sanger sequencing showed germline nonsense mutation of *ARMC5* c.967C>T (p.Gln323Ter). The second somatic missense mutation of *ARMC5* was detected in one out of two resected nodules, reflecting the second-hit model of tumorigenesis. Routine genetic testing in his apparently healthy offspring showed one of two daughters and one son harbored the germline mutation.

**Conclusions:**

In conclusion, our case report highlight the importance of genetic testing in the molecular diagnosis of PBMAH. Genetic screening in related family members will find out asymptomatic variant carriers to guide life-long follow-up.

**Supplementary Information:**

The online version contains supplementary material available at 10.1186/s12920-021-00896-0.

## Background

Primary bilateral macronodular adrenocortical hyperplasia (PBMAH), also known as ACTH-independent macronodular adrenal hyperplasia (AIMAH), is a rare form of Cushing’s syndrome (CS). It has been reported that PBMAH accounts for 6.2–9.0% of all endogenous causes of CS patients in China [1, 2]. PBMAH was firstly described by Kirschner MA et al. in a patient presenting with long-standing CS and bilateral multinodular adrenal hyperplasia [3]. Typical signs by CT examination showed multiple nodules in bilateral adrenal glands with characteristic “a bunch of grapes appearance” [4]. Substantial clinical heterogeneity has been observed in PBMAH, ranging from subclinical CS without visible symptoms to overt CS with severe complications. This disease is most frequently diagnosed in patients between the fourth and sixth decades of life [2], which is much older than most patients with CS caused by unilateral adrenocortical tumors. Bilateral nodules gradually grow in a polycentric manner, indicating that genetic predisposition and acquired pathophysiological factors are involved in disease progression.

Several molecular mechanisms have been demonstrated to be participated in the process of aberrant nodule formation. One common type of PBMAH is caused by ectopic adrenocorticotropic hormone (ACTH) secretion from steroidogenic cells disseminated within adrenal glands [5]. Thus, the nomenclature, PBMAH, seems to be more suitable than the term AIMAH. PBMAH are usually caused by inactivating mutations in a number of tumor suppressor genes, such as menin [6–8], fumarate hydratase [9], adenomatous polyposis coli gene (*APC*) [10] and armadillo repeat-containing protein 5 (*ARMC5*) [11]. Relatively rare forms of PBMAH include activating mutations in ACTH receptor gene and gene mutation leading to constitutive activation of cAMP/protein kinase A (PKA) signaling pathway, including guanine nucleotide-binding protein alpha-stimulating activity polypeptide (*GNAS*), cAMP-dependent protein kinase catalytic subunit alpha (*PRKACA*), phosphodiesterase 11A (*PDE11A*) and phosphodiesterase 8B (*PDE8B*)[12]. Although the pathophysiological processes are not fully understood, about 20% to 50% cases are observed to harbor the inactivating mutations of *ARMC5* [13–15]. Herein, we have uncovered a novel nonsense mutation of *ARMC5* in a Chinese patient diagnosed with sporadic PBMAH.

This patient had been misdiagnosed as primary hypertension for 5 years and type 2 diabetes mellitus for 11 years, for insidiously progressed symptoms of CS had been atypical over many years. The etiology was not identified until the finding of adrenal masses because of resistant hypertention. We have routinely performed gene sequencing for *ARMC5* in the patient and his offsprings. A novel germline mutation and a novel somatic mutation of *ARMC5* were detected in the patient. Two of his three offsprings were found to be asymptomatic carriers.

## Case presentation

### A CS patient with bilateral adrenal masses

**Fig. 1:**
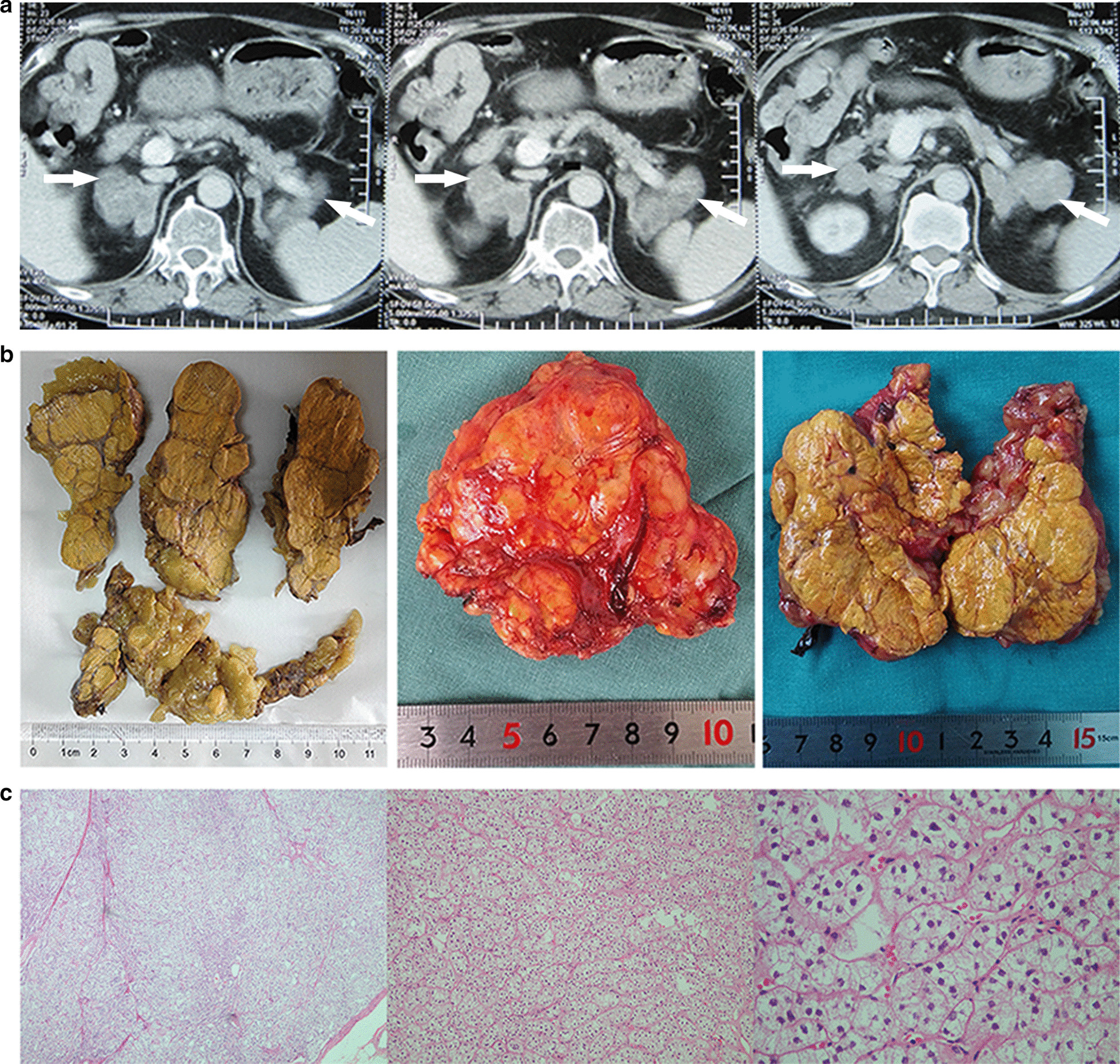
Clinical data of the proband. **a** consecutive abdominal CT images showed bilateral irregular adrenal masses. **b** Multiple nodules in different size (over 1 cm) with “grapes appearance” were observed in the adrenal glands (left: cross-sectional view of formaldehyde-fixed right adrenal gland, middle: gross view of left adrenal gland, right: cross-sectional view of left adrenal gland). **c** Hematoxylin–eosin (HE) staining of resected tissues showed diffuse hyperplasia of zona fasciculate (from left to right: magnification, × 40, × 100, × 400)

**Fig. 2:**
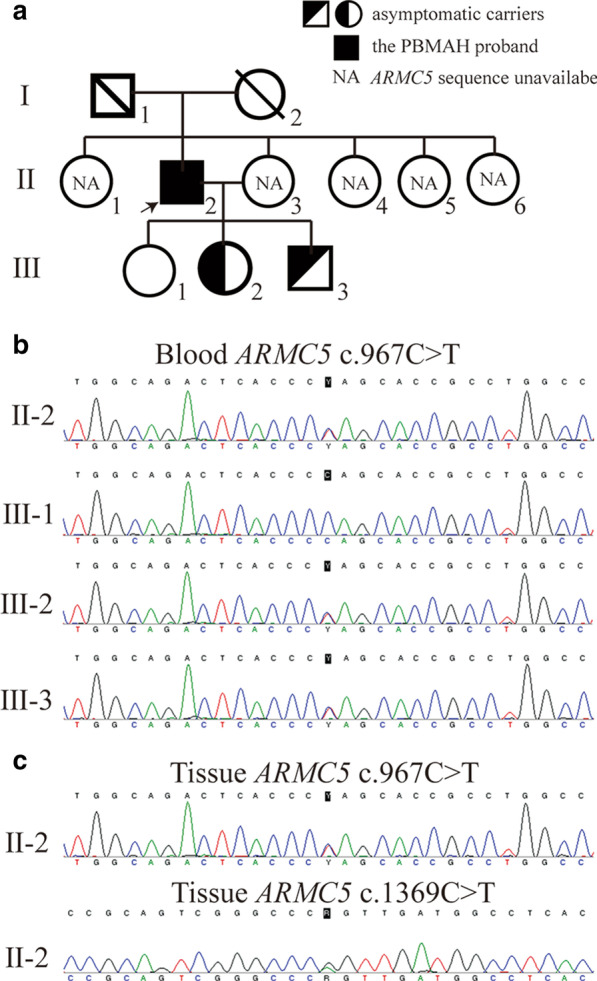
Genetic diagnosis of PBMAH in the proband and his offspring. **a** Family tree of the patient. The arrow indicates the proband. **b** Sanger sequencing of *ARMC5* in peripheral blood samples of the patient and his offspring. **c** The second mutation (c.1369C>T) was identified from one out of two resected nodules

A 51-year-old Chinese man suspected of secondary hypertension was admitted for poorly controlled blood pressure and bilateral adrenal masses. He had a six-year history of hypertension and was treated with valsartan (80 mg) and metoprolol (47.5 mg). One month before the admission, he complained of puffiness of the face and uncontrolled blood pressure to his local physician. The blood pressure was 205/115 mmHg. Laboratory test showed K^+^ 2.71–3.73 mmol/L (decreased), HCO3^−^ 33.3–33.8 mmol/L (increased) and elevated aldosterone renin ratio (ARR) (Table 1), indicating chronic hypokalemia correlated with hyperaldosteronism. After he had been treated with valsartan (80 mg), amlodipine (5 mg), metoprolol (47.5 mg) and spironolactone (60 mg), the blood pressure was 170–180/114–120 mmHg. The CT-scanning in adrenal glands showed bilateral enlargement of adrenal glands (Fig. 1a). So, he was referred to our center for further examination and treatment. On admission, he was 65 kg in weight, 165 cm in height, with a body mass index at 23.9 kg/m^2^. He had eleven-year history of diabetes mellitus (DM). He got desirable glucose control treated by twice-daily premixed insulin plus oral antidiabetic agents. He also had one-year history of spinal compression fracture. No family history of endocrine diseases was remarkable for him. His father (I1, Fig. 2a), who died of acute cerebral stroke at the age of 72, had hypertension and DM at the age of 62 and 70, respectively. His mother died in an accident at the age of 30. His elder sister (II1, Fig. 2a) was diagnosed as DM and hypertension several years ago. She had desirable control of DM and blood pressure with oral agents. His other three sisters were apparently healthy. The tests for cortisol levels in his sisters were all normal at local hospital. Physical examination showed signs of Cushing’s syndrome, including facial flushing, plethora, skin bruisability, centripedal obesity, supraclavicular fat pad and buffalo hump. Z-score of lumbar vertebrae (L1–L4) and femoral neck was − 1.6 and − 1.5 on Dual energy X-ray absorptiometry scan, respectively, indicating osteopenia for his age.

**Table 1:** The main laboratory and dexamethasone suppression test results

Test	Result	Reference (unit)
WBC	8.03	4–10 (*10^9^/L)
Hemaglobulin	128	120–160 (g/L)
Platelet	141	100–300 (*10^9^/L)
Urinary protein	±	Negative
ALT	21	4–41 (U/L)
AST	15	4–40 (U/L)
Creatinine	85	59–104 (μmol/L)
Uric acid	453.4 ↑	202.3–416.5 (μmol/L)
eGFR	91	> 90 (ml/min/1.73 m^2^)
HCO_3_^−^	33.3–33.8 ↑	22–29 (mmol/L)
K^+^	2.71–3.73 (low)	3.50–5.10 (mmol/L)
FPG	6.09	3.90–6.10 (mmol/L)
HbA1c	7.3% ↑	4.40–6.50%
TSH	0.71	0.27–4.20 (μIU/L)
hsCRP	2.90 ↑	0–1 (mg/L)
NT-proBNP	654 ↑	83.9 (pg/ml)
Renin	0.77	0.10–6.56 (ng/ml/hour)
Aldosterone	23.75	7–30 (ng/dl)
ARR	30.84 ↑	< 25
Adrenaline	1.86	1.21–2.30 (nmol/L)
Noradrenaline	2.20	1.31–2.51 (nmol/L)
ACTH	1.63 (low)	1.60–13.9 (pmol/L)
Cortisol (8AM)	332.20 ↑	62–194 (μg/L)
Cortisol (4PM)	245.20 ↑	23–119 (μg/L)
Cortisol (12MN)	295.20 ↑	
Free cortisol in 24 h urine	848.24 ↑	36–137 (μg/24 h)
LDDST: cortisol (8AM)	275.00 ↑	< 18 (μg/L)
HDDST: cortisol (8AM)	239.60 ↑	

*WBC* white blood cell, *ALT* alanine aminotransaminase, *AST* aspertate aminotransferase, *eGFR* estimated glomerular filtration rate, *FPG* fasting plasma glucose, *HbA1c* Hemoglobin A1c, *TSH* thyroid stimulating hormone, *hsCRP* hypersensitivity C-reactive protein, *NT-proBNP* N-terminal prohormone of brain natriuretic peptide, *ARR* aldosterone renin ratio, *ACTH* adrenocorticotropic hormone, *AM* ante meridiem, *PM* post meridiem, *MN* midnight, *LDDST* low-dose dexamethasone suppression test (1 mg overnight), *HDDST* high-dose dexamethasone suppression test (8 mg/day for 2 days), ↑ the arrow denotes the results above the upper limit of normal

### Molecular diagnosis of PBMAH

Considering the high risk for relapse of CS after partial adrenalectomy, bilateral adrenalectomy was chosen. His symptoms and signs had been gradually relieved after removal of bilateral adrenal glands. The resected adrenal glands were shown (Fig. 1b, c). Considering *ARMC5* inactivation is the most common etiology of PBMAH, we performed genetic analysis of *ARMC5* gene both from the peripheral blood and two adrenal nodules to detect germline and somatic mutations, separately. Family pedigree was drawn (Fig. 2a). Coding exons and adjacent splice junctions were amplified for the *ARMC5* gene. Sanger sequencing was carried out bi-directionally on ABI 3500 Dx. The information on PCR primers used for amplication was shown in Additional file 1: Table S1. NM_001288767 was used as a reference sequence of *ARMC5*. We detected *ARMC5* germline mutation at c.967C>T, leading to premature termination (p.Gln323Ter). The second mutation c.1369C>T (p.Arg457Trp) was identified in one out of two nodules from the right adrenal gland (Fig. 2c). In silico predictions on functional impacts using SIFT, Polyphen2, CADD and GenoCanyon suggested that the substitution may be damaging, which were summarized in VarCards (http://varcards.biols.ac.cn/) (Additional file 2: Table S2). Six months after the operation, he got desirable glucose control with oral hypoglycemic agents plus basal insulin.

His blood pressure was controlled to normal range only with nifedipine (30 mg). Hydrocortisone (20 mg) and flurocortisone (0.1 mg) replacement therapy was used to substitute normal adrenal function. Genetic screening was performed in his two daughters and one son. Unfortunately, his younger daughter (III2) and son (III3) carried the same *ARMC5* germline mutation (Fig. 2b), although they were apparently healthy.

## Discussion and conclusions

The majority of primary adrenal CS is caused by benign unilateral adenoma, accounting for 75–90% of all cases. Less than 5% of patients are attributed to adrenocortical carcinoma. Approximately 10% of the lesions are caused by bilateral adrenal hyperplasia, of which PBMAH belongs to the commonest form [16]. The involvement of bilateral adrenal glands in PBMAH indicates genetic causes play critical roles in the pathogenesis. In recent years, it has been demonstrated that the genetic etiology of PBMAH is caused by aberrant activation of cAMP/PKA pathway or inactivation of *ARMC5*.

Assie et al. have employed genome-wide approaches to identify the germline inactivation of *ARMC5* as widely existing phenomenon in PBMAH patients [11]. In all patients harboring germline *ARMC5* mutation, the second mutation on the somatic level was detected in the resected nodules. ARMC5 is a cytosolic protein with largely unknown functions. ARMC5 may interact with several proteins to regulate fetal development, cell proliferation and apoptosis [17]. Recently, it has been demonstrated that ARMC5 degradation could be mediated by the ubiquitin–proteasome system through interaction with Cullin3. The degradation of ARMC5 has been shown to be beneficial for cell proliferation, indicating it has tumor suppressor effect [18]. Remarkable embryonic lethality was observed in *ARMC5* knockout (*ARMC5*^−/−^) mice [17, 19], in those who survived the sizes of adrenal glands were normal at birth while enlarged at older age (> 15 months) [17]. *ARMC5* heterozygote mice (*ARMC5*^+/−^) showed decreased corticosterone levels at 1 year while presented with hypercorticosteronemia at 1.5 years old. These manifestations were much close to PBMAH patients with *ARMC5* haploinsufficiency. Current data support the notion that the overall glucocorticoid secretion gradually progresses as the advantage in cell numbers compensates the inefficient steroidogenesis per cell. The insidious occurrence of mild hypercorticosteronemia renders timely diagnosis of PBMAH to be difficult. Consequently, they have mild diabetes mellitus and hypertension which progress gradually with long-term misdiagnosis. For this case, the patient suffered from 11-year history of diabetes mellitus and 6-year history of hypertension. He also had compressed spinal fracture and suspected kidney impairment with trace urinary protein. Earlier diagnosis of PBMAH may prevent the long-term complication of CS. According to the peak age of disease onset, the fourth to sixth decade of life should be monitored strictly in *ARMC5* pathogenic mutation carriers.

ARMC5 belongs to Armadillo (ARM)-containing protein family, characterized by the presence of tandem repeats of ARM motif composed by 42 amino acids [20]. The nomenclature “Armadillo” is derived from the appearance of fly embryos with mutated Drosophila segment polarity gene *armadillo* [21], well-known as a mammalian ortholog, β-catenin. Another ARM-containing protein, namely APC, plays a role in canonical wingless/integration-1 (Wnt) signaling [22]. Remarkably, ARMC5, β-catenin and APC are all involved in the adrenal tumorigenesis in PBMAH [5, 10, 23]. The ARM domain offers highly conserved spacial architecture for protein–protein interactions [20]. Thus, it is not striking that ARMC5 has multiple functions encompassing but not limited to embryonic development, pro-apoptosis, regulation of cortisol synthesis and certain ectopic GPCRs expression in steroidogenic cells in zona fasciculata [11, 13, 17, 24]. Activation of ectopic GPCRs and ACTH receptors shares the common cAMP/PKA signaling pathway, culminating in similar growth-promoting effects on steroidogenic cells. Complete remission was observed in a majority of patients who utilize pharmacological therapies targeting against aberrantly expressed GPCRs [25]. For most PBMAH cases, responsiveness to agents appears to be partial, transient or completely failed [26]. Normally, two or more GPCRs were simultaneously expressed on adrenocortical cells, rendering difficulties in limited drug therapy. Some patients could benefit from combined therapy of unilateral adrenalectomy and targeted GPCR antagonists [27]. For this case, we performed bilateral adrenalectomy considering that the patient had overt CS symptoms and markedly enlarged adrenal masses on both sides. The cellular events in adrenal gland caused by *ARMC5* mutation are multifaceted, resulting in not only dedifferentiation but also proliferation of steroidogenic cells. Thus, it seems that pharmacological intervention against one or two of multiple abnormalities secondary to *ARMC5* mutation, such as using antagonists to block ectopic GPCRs activation, is not likely to fully impede the pathophysiological process. Nevertheless, it is suitable to use targeted agents for patients at early stage of PBMAH. In this patient, whether ectopic GPCRs were expressed in resected nodules remained unknown. Theoretically, targeted genetic therapy to correct the mutation appears to be the ideal choice while it is unavailable both in fetal and adult individuals. It should be pointed out that pathogenic *ARMC5* mutation carriers are not definitely developing to PBMAH [19, 28], not only in mice models but also in clinical settings. Different patients with pathogenic *ARMC5* mutations have broad spectrum of manifestation, from normal cortisol secretion, mildly subclinical CS to overt CS [28]. The heterogeneities among different individuals indicate that types of *ARMC5* mutation, genetically driving force of the second-hit on *ARMC5*, age and other unknown factors may be involved in the disease onset.

The germline mutation of *ARMC5* in the proband results in premature termination at the 322nd amino acid residue located in the ARM domain. This mutation presumably leads to inactivation of ARMC5. In our study, DNA samples were derived from two isolated nodules. We found a missense mutation (p.Arg457Trp) in one of two nodules. Several in silico prediction softwares support that the mutation is damaging (Additional file 2: Table S2). No visible mutation could be detected in the coding sequence of *ARMC5* in another nodule. It seems that the negative finding on somatic mutation contradicts the second-hit hypothesis. However, we should note that the limitation of Sanger sequencing used here to detect somatic mutation may be the reason for the negative finding. The somatic mosaicism in nodules might be missed by Sanger sequencing which has mutation detection limit around 20% [29]. Independent somatic mutations are likely to be ascribed to accumulated genetic events during cell proliferation [30] or intrinsic instability of the locus. It remains unknown whether PBMAH could be caused by concomitant heterozygous mutation of other gene(s) combined with *ARMC5* germline mutation.

The surgical strategies of PBMAH include unilateral, bilateral and subtotal adrenalectomy. A unilateral procedure might not yield a cure and requires contralateral adrenalectomy later in the lifetime [31, 32]. Unilateral adrenalectomy has been shown to be effective with a low recurrence rate in PBMAH patients under a five-year follow-up [33]. Complete resection of both adrenal glands makes hormone replacement to be a life-long issue. Thus, subtotal adrenalectomy to reduce the number of adrenocortical cells may be a choice to cure PBMAH. However, it remains unknown how much proportion of adrenal tissues should be reserved for each individual.

In this patient, the aldosterone renin ratio was marginally increased. Uncorrected hypokalemia due to hypercortisolism, antihypertensives, such as valsartan and metoprolol, are well-known to negatively regulate the secretion of aldosterone. Thus, the level of aldosterone present here didn’t realistically reflect the status before intervention with agents. We could not exclude the possibility that this patient had concurrent primary hyperaldosteronism (PA). The relationship between pathogenic *ARMC5* mutation and PA remains to be investigated. Although serum aldosterone levels were not altered in *ARMC5*^+/−^ mice at different time points, β-catenin accumulation in nucleus of glomerulosa cells was slightly increased in these mice [19]. The activation of Wnt/β-catenin signaling has been documented to be involved in the secretion of aldosterone in aldosterone-producing adenomas [34]. At the same time, aldosterone levels also mildly increased due to expansion of the adrenocortical steroidogenesis cells. However, *ARMC5* mutation was not consistently associated with PA in clinical settings [35, 36].

One suspending issue is why ARMC5 is widely expressed while its mutation normally affects adrenal glands [37]. Actually, ARMC5 expression has been shown to be higher in the brain, spinal cord, lymphocytes, adipose tissue than in the adrenal gland. Concomitant PBMAH and meningiomas have been observed a second-hit mutation of *ARMC5* in haploinsufficiency carriers [38, 39]. Certain PBMAH cases were reported to have concomitant primary hyperparathyroidism [40, 41]. *ARMC5* deficiency has also been associated with compromised T-cell immune responses in mice models [17]. Therefore, for patients with *ARMC5* mutation, systematic evaluation of risks on PBMAH, meningiomas, hyperparathyroidism and hyperparathyroidism, PA and other tumors seems necessary.

However, there are several limitations in our case report. Firstly, the inheritance pattern of *ARMC5* mutation in the proband’s whole family members was unknown. His mother, father and siblings were probably asymptomatic carriers or overt patients. Secondly, we have merely performed the gene sequencing of *ARMC5*. As we mentioned previously, several genes are involved in the pathogenesis of PBMAH. The absence of *ARMC5* mutation could not exclude the possibility that other genes are responsible for the disease onset. So, next-generation sequencing techniques encompassing all the possible pathogenic genes have great advantage in genetic diagnosis. Thirdly, the somatic mutation was not unanimously predicted as pathogenic using different in silico prediction softwares. Functional assessments are warranted to ascertain the relationship between genotype and phenotype. Fourthly, it remains open that subtotal resection of adrenal glands could improve the patient’s life quality. The patient underwent hyperkalemia when he was treated with hydrocortisone alone. So, he had to take flurocortisone to treat the hypoaldsteroidism.

In conclusion, our case report highlight the importance of genetic testing in the diagnosis of PBMAH. Genetic screening in related family members will find out asymptomatic variant carriers to guide life-long follow-up.

## Supplementary Information

**Additional file 1: Table S1.** Primers used to amplify the exons of *ARMC5*.

**Additional file 2: Table S2.** In silico predictions of *ARMC5* c.1369C>T (p.Arg457Trp) on functional impacts using VarCards (http://varcards.biols.ac.cn/).

## Data Availability

All data generated or analyzed in this study are included in this manuscript.

## Abbreviations

PBMAH: Primary bilateral macronodular adrenocortical hyperplasia
ARMC5: Armadillo repeat-containing protein 5
ACTH: Adrenocorticotropic hormone
AIMAH: ACTH-independent macronodular adrenal hyperplasia
CS: Cushing’s syndrome
APC: Adenomatous polyposis coli gene
PKA: Protein kinase A
GNAS: Guanine nucleotide-binding protein alpha-stimulating activity polypeptide
PRKACA: cAMP-dependent protein kinase catalytic subunit alpha
PDE11A: Phosphodiesterase 11A
PDE8B: Phosphodiesterase 8B
ARR: Aldosterone renin ratio
DM: Diabetes mellitus
HE: Hematoxylin–eosin
ARM: Armadillo
Wnt: Wingless/integration-1
GPCRs: G protein-coupled receptors
