# Supplementary material for: A novel nonsense mutation in ARMC5 causes primary bilateral macronodular adrenocortical hyperplasia
Source: BMC Med Genomics. 2021 May 10;14:126. doi: 10.1186/s12920-021-00896-0 (PMC8108324; doi:10.1186/s12920-021-00896-0)
Supplement: Supplementary file 1 — Additional file 1: Table S1. Primers used to amplify the exons of ARMC5. [file 12920_2021_896_MOESM1_ESM.docx]

| No. | Primer Name | Primer sequence(5' to 3') |
| --- | --- | --- |
| 1 | ARMC5 E1F (536 bp) | CCGCAGCAGATTCCAACT |
| 2 | ARMC5 E1R (536 bp) | AGCAGCCCTTCCTGGTGT |
| 3 | ARMC5 E2F (505 bp) | GAGCCACCTCGCAATCCA |
| 4 | ARMC5 E2R (505 bp) | CCGAACAGCCCAGAAGTCC |
| 5 | ARMC5 E3-1F (498 bp) | GCTTCCCGAGGTAGGCGTGAGA |
| 6 | ARMC5 E3-1R (498 bp) | CGCCGTAGCAGCGCGAGTAG |
| 7 | ARMC5 E3-2F (622 bp) | AGGACCCAGCGACCAACGA |
| 8 | ARMC5 E3-2R (622 bp) | GTCCTCAACAACCAATTCCT |
| 9 | ARMC5 E4F (401 bp) | CCCTGCCGACCATTAGCCT |
| 10 | ARMC5 E4R (401 bp) | ACGCACCACGCTCTGTAGGC |
| 11 | ARMC5 E5-1F (593 bp) | TGGGGAACTTAGCCATGGAA |
| 12 | ARMC5 E5-1R (593 bp) | GGCTAGCTCCATTAGGATCCC |
| 13 | ARMC5 E5-2F (633 bp) | GCAGTACGCGAGGGAACCAT |
| 14 | ARMC5 E5-2R (633 bp) | GCAGGTGGTCGTGTGGGAAT |
| 15 | ARMC5 E6F (679 bp) | TCTGCCCCTTAACCTTGGCT |
| 16 | ARMC5 E6R (679 bp) | ACAGGTTGTCTGGAGGACGG |
| 17 | ARMC5 E7F (482 bp) | TTCAGTGCCCTGATTTCTCA |
| 18 | ARMC5 E7R (482 bp) | GGAGGCAGGAAAGGGAGT |
| 19 | ARMC5 E8-1F (584 bp) | GCCCTGAGGACCGAGTGGC |
| 20 | ARMC5 E8-1R (584 bp) | CCCCCGACAACCATGCAAAT |
| 21 | ARMC5 E8-2F (634 bp) | GCCACCGCCTCCCCTTTCT |
| 22 | ARMC5 E8-2R (634 bp) | CGGTTCCATGTTCTCACCGCT |
| Note: E denotes exon. Forward (F) and reverse (R) primers are shown. | | |

Table S1. Primers used to amplify the exons of *ARMC5*
